# Time-domain analysis of scrotal thermoregulatory impairment in varicocele

**DOI:** 10.3389/fphys.2014.00342

**Published:** 2014-09-16

**Authors:** Enas Ismail, Giuseppe Orlando, Paolo Pompa, Daniela Gabrielli, Luigino Di Donato, Daniela Cardone, Arcangelo Merla

**Affiliations:** ^1^Department of Neuroscience, Imaging and Clinical Sciences, Gabriele d'Annunzio University of Chieti PescaraChieti-Pescara, Italy; ^2^Institute for Advanced Biomedical Technologies (ITAB), G. d'Annunzio UniversityChieti-Pescara, Italy; ^3^DIIGA, Polytechnic University of MarcheAncona, Italy; ^4^Department of Urology, Ospedale CivilePescara, Italy

**Keywords:** control system, modeling, multinomial logistic regression, Functional Infrared (fIR), scrotal temperature, scrotal thermoregulation, varicocele

## Abstract

Varicocele is a common male disease defined as the pathological dilatation of the pampiniform plexus and scrotal veins with venous blood reflux. Varicocele usually impairs the scrotal thermoregulation via a hemodynamic alteration, thus inducing an increase in cutaneous temperature. The investigation of altered scrotal thermoregulation by means of thermal infrared imaging has been proved to be useful in the study of the functional thermal impairment. In this study, we use the Control System Theory to analyze the time-domain dynamics of the scrotal thermoregulation in response to a mild cold challenge. Four standard time-domain dynamic parameters of a prototype second order control system (Delay Time, Rise Time, closed poles locations, steady state error) and the static basal temperatures were directly estimated from thermal recovery curves. Thermal infrared imaging data from 31 healthy controls (HCS) and 95 varicocele patients were processed. True-positive predictions, by comparison with standard echo color Doppler findings, higher than 87% were achieved into the proper classification of the disease stage. The proposed approach could help to understand at which specific level the presence of the disease impacts the scrotal thermoregulation, which is also involved into normal spermatogenesis process.

## 1. Introduction

Varicocele is defined as the pathological dilatation of the pampiniform plexus and of the scrotal veins with venous blood reflux (Herman, 1975). Varicocele may cause subfertility or infertility and testicular pain (Masson and Brannigan, 2014). It is present in 15% of the adult male population, in 35% of men with primary infertility, and in 80% of men with secondary infertility (Romeo and Santoro, 2009). Varicocele has been observed to be predominantly a left-sided lesion (Kaufman and Nagler, 1986). Ninety percent incidence of isolated left-sided clinical varicocele was reported (Saypol, 1981), even though recent studies have indicated that bilateral varicocele may be much more common than previously appreciated (Gat et al., 2004a,b; Canales et al., 2005). Left varicocele is more commonly found because the length of the left internal spermatic vein is longer than the right spermatic vein (Kaufman and Nagler, 1986). Additionally, the left internal spermatic vein enters the left renal vein perpendicularly; while on the right, the internal spermatic vein drains obliquely into the inferior vena cava. These combined anatomic features may result into increased hydrostatic pressure transmitted to the venous drainage system of the left testicle, thus resulting in the occurrence of venous dilatation and varicocele formation (Nagler and Grotas, 2009). Varicocele can be diagnosed by Echo Color Doppler imaging and categorized in five grades according to Pauroso et al. (2011) (see Table 1).

**Table 1:** **Echo Color Doppler classification for varicocele**.

**Classification**	**Grade**	**Features**
vr1	Grade I	Prolonged reflux in the vessels of the inguina canal only during the Valsalva maneuver.
	Grade II	Small posterior varicosities reach the upper pole of the testicle and increase in diameter during the Valsalva maneuver.
vr2	Grade III	Vessels appear dilated up to the lower pole of the testicle when the patient is standing upright, whereas no dilatation is evident with the patient in the supine position. Color Doppler shows evident reflux only during the Valsalva maneuver.
vr3	Grade IV	Venous dilatation identifiable with the patient both standing and supine. The dilatation increases with the patient standing up and during the Valsalva maneuver.
	Grade V	Presence of patent venous dilatation in both the prone and supine position. Color Doppler shows significant baseline venous reflux which does not increase after the Valsalva maneuver.

As for the clinic, varicocele can be diagnosed through palpation of the scrotum and classified as reported in Dubin and Amelar (1970) (see Table 2).

**Table 2:** **Clinical classification for varicocele**.

**Grade**	**Features**
Subclinical	Non-palpable enlargement of the venous plexus of the spermatic tone, which is diagnosed only by ultrasound, angiography, or any other imaging method.
Grade I	Small palpable distensions detected only during a Valsalva maneuver.
Grade II	Moderate with easily palpable distension on upright examination.
Grade III	Large visible veins on upright examination without palpation.
Grade IV	Very large varicosities become visible immediately when the patient stands up; the varicosities are hypertensive, and subcutaneous varices are present too.

Varicocele impairs scrotal thermoregulation with resultant increase in testicular temperature (Kaufman and Nagler, 1986), as shown by a series of studies based on the evaluation of scrotal cutaneous temperature through thermal infrared (IR) imaging (Merla et al., 2002c; Watanabe, 2002; Gat et al., 2004a; Merla et al., 2004; Nogueira et al., 2009). In normal men, the testicular temperature is from 3^o^ to 4^o^C lower than core body temperature (Mieusset and Bujan., 1995; Thonneau et al., 1998; Romeo and Santoro, 2009). Two main thermoregulatory processes control the testicular temperature: heat exchange with the environment through the scrotal skin and heat clearance by blood flow through the pampiniform plexus (Thonneau et al., 1998; Masson and Brannigan, 2014). In particular, it has been demonstrated that, apart from the presence of hyperthermia, the affected testicle recovers faster from a mild cold stress with respect to the healthy one (Merla et al., 2002c, 2004; Mariotti et al., 2010). Specifically, on the basis of the heat-balance equation, the re-warming processes of the affected scrotum at the level of the testicle site (t) and of the pampiniform plexus (p), i.e., the proximal portion of the veins draining the scrotal blood, were dynamically characterized by shorter time constant (the recovery time needed to return to the pre-cooling temperature) τ_t_ and τ_p_, and by an augmented differences between left and right testicles Δτ_t_ and Δτ_p_ (Merla et al., 2001, 2002b,c, 2004). Alternative approaches have been proposed to model the scrotal cutaneous thermoregulation on the basis of the automatic control theory. With an open-loop analysis, Sealfon and Zorgniotti (1991) suggested that in human testis there is no feedback or regulation, indicating that any internal or external factor causing a temperature change will not trigger a feedback mechanism to control the testis temperature. Conversely, Mariotti et al. (2010) highlighted the possibility of evaluating the scrotal thermoregulatory impairment through automatic control theory on the basis of its homeostatic negative feedback loop. However, the association between the model components with the local active rewarming and passive heat exchange remains hypothetical and speculative, given the elevated complexity of the system and the need for a better understanding of the correspondence of the model to the actual physiological processes. Based on the previously published evidence that the skin thermoregulatory system could be modeled as a second order system (Rollins et al., 2006), Ismail et al. (2014) demonstrated that a direct estimation of the closed loop step dynamic response parameters based on time domain analysis could provide an effective description of the functional differences among patients and healthy controls in finger thermoregulation, supporting a proper differential diagnosis of the disease based on thermal imaging data and the assessment of the functional impairment after thermal stimulation. Therefore, in order to describe the time domain dynamics of scrotal thermoregulation in response to a standard cold challenge, we adopted a prototype second order control system as a model and proposed to directly estimate its standard time-domain specifications. In addition, we also evaluate the subject classification capability of our method in order to distinguish healthy from left-sided varicocele and to assess its diagnostic specificity.

## 2. Scrotal thermoregulatory system

**Figure 1:**
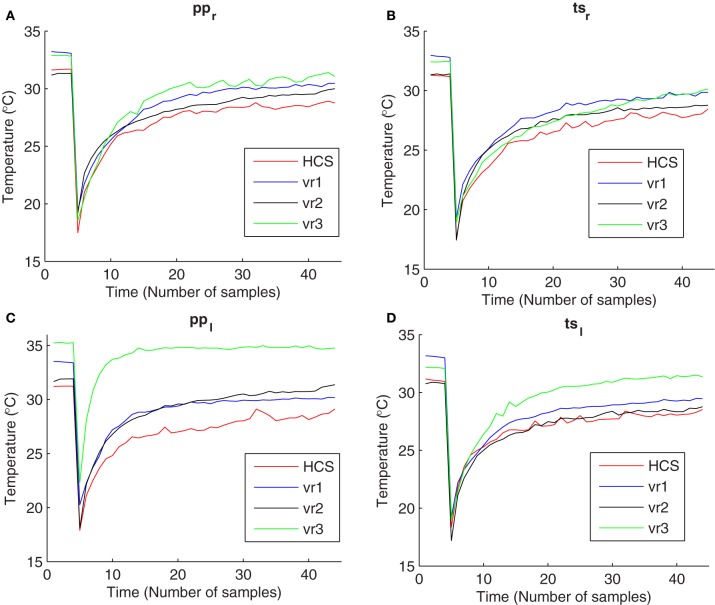
**Temperature vs. time curves obtained from thermal imaging data during cold stress test for Healthy Controls (HCS), varicocele grade I and II (vr1), varicocele grade III (vr2), and varicocele grades IV and V (vr3) at four regions of interest: (A) pampiniform plexus in the right scrotum (pp_r_), (B) testicular site in the right scrotum (ts_r_), (C) pampiniform plexus in the left scrotum (pp_l_), and (D) testicular site in the left scrotum (ts_l_)**.

Scrotal thermoregulation serves to liberate the large amount of heat produced during spermatogenesis. A number of supporting mechanisms like thin skin with abundant vascularization, numerous sweat glands, and absence of subcutaneous fat facilitates heat exchange and contributes to maintain the testicular temperature below body temperature (Skandhan and Rajahariprasad, 2007). Venous stasis associated with varicocele increases cutaneous temperature of the affected testicle or pampiniform plexus (Merla et al., 2002c). Exposure to cold stress elicits cutaneous vasoconstriction, accompanied by increased skin rugosity to reduce the surface area involved into heat exchange with the environment (Sawasakia et al., 2001). After the cessation of cold exposure, homeostatic processes restore basal pre-stress conditions, mostly through vasodilatation, favoring heat exchange with deeper layers (Merla et al., 2002a). In the presence of varicocele, affected testicles return to pre-stress equilibrium temperature faster than normal testicles (Merla et al., 2002c) (Figure 1).

Homeostasis is basically maintained by a negative feedback loop, similar to a thermostat (Sanial and Maj, 2001) which regulates the energy exchange with the environment at the cutaneous level through metabolic and hemodynamic processes that determine the cutaneous temperature at any given time (Rene et al., 1995). From the control system theory, the basic functional components of homeostasis can be thought of as arranged in a feedback loop: a controlled plant (the scrotal thermal processes), whose output (the cutaneous scrotal temperature) is constrained to follow a given set-point (the reference basal value) through an internal feedback loop (homeostatic mechanism). The cutaneous basal temperature can be considered the reference value that is assumed to be almost constant (like a step signal). To study the system dynamics, the system has to be stimulated by a proper functional input. The process of studying the recovery patterns is defined by control system theory as time-domain analysis of the dynamic response. Namely, a cold challenge induces a scrotal cutaneous temperature (system dynamic output response) change from the basal value (reference signal). The cold stress induces a variation from basal value of the controlled output. In feedback systems, the output signal is compared with the reference value thus generating an error signal, which stimulates the thermoregulatory control reaction in order to restore the basal value. In other words, the thermoregulatory reaction steers the error to zero.

The time evolution of scrotal temperature can be recorded by means of thermal IR imaging (Mariotti et al., 2010). An example of temperature vs. time curves obtained from experimental recovery data are reported in Figure 1. The thermoregulatory system of the scrotal skin was demonstrated to be a second-order time-invariant system with exponential critically-damped dynamic response (Rene et al., 1995; Merla et al., 2002b). The recovery curves of the temperature can be interpreted, according to the control system theory, as the feedback system response to a perturbation of the operative conditions. Differences in the recovery curves depend on the capability of the control system to recover. By studying the time-domain characteristics of the dynamic responses shown by the recovery curves, we can find useful insights about the actual values of functional modeling parameters.

## 3. Materials and methods

### 3.1. Subjects

Ninety-five varicocele patients and 31 healthy controls (HCS) participated in this study, which was authorized by the Human Board Review and Local Ethical Committee of the School of Medicine of the University of Chieti-Pescara. None of the subjects has already had sons before participating into the study. All participants provided written informed consent to participate in the study, which was approved by the local Ethical Committee and Institutional Review Board. Participants were excluded if they presented any genital disease other than left varicocele or history of genital diseases, cigarette smoking, cardiovascular, or neurovascular disorders, hypertension, history of drug or alcohol abuse, and any therapeutic treatment. Patients suffering from right or bilateral varicocele and/or hydrocele were excluded as well to avoid possible confounding results. Demographic data of the participating subjects are summarized in Table 3. According to Echo Color Doppler investigation, the 95 patients were classified as: 32 vr1 (echo color Dopplergrade I and II), 35 vr2 (echo color Doppler grade III), 28 vr3 (echo color Doppler grade IV and V).

**Table 3:** **Clinical classification for varicocele**.

**Item**	**HCS**	**vr1**	**vr2**	**vr3**
Description	Healthy controls	Grade I-II	Grade III	Grade IV-V
No. of subjects (125)	31	32	35	28
Age (Mean ± Std) (Years)	37 ± 4	36 ± 4	36 ± 6	33 ± 8

### 3.2. Data collection

Participants underwent to clinical examination and echo color Doppler imaging (ATL 5000 echo color Doppler imaging system, Philips Medical System, Eindhoven, The Netherlands). For each subject, the functional response to a mild cold challenge of scrotum was assessed by thermal IR imaging (Merla et al., 2002c, 2004). All participants were asked to refrain from physical activities and intake of vasoactive substances for 2 h prior to the measurements. Before undergoing measurements, the subjects took off their pants and underwear leaving exposed only the scrotum, the penis and the thigh. Then they moved to the recording room, which was set at a standardized temperature (23°C), humidity (50–60%), and without direct ventilation, in which they underwent to 20-min acclimatization period prior to undergoing the thermal imaging. The subjects sat comfortably during both acclimatization and measurement periods and were asked to keep their legs slightly divaricated in order to facilitate the thermal IR imaging (Merla et al., 2002c, 2004). The penis was gently attached to the lower abdomen by using medical tape in order to obtain clear thermal images of the scrotum. Thermal IR imaging was performed by means of a digital thermal camera (FLIR SC3000, FlirSystems, Sweden), with a Focal Plane Array of 320 × 240 QWIP detectors, capable of collecting the thermal radiation in the 8–9 μm band, with a 0.02 s time resolution, and 0.02 κ temperature sensitivity. Cutaneous emissivity was assumed as ε ≈ 0.98 (Merla et al., 2002c). Thermal images of the scrotum of each subject were recorded for 25 min, acquiring images every 30 s. Five thermal images were recorded before the cold stress, (scrotal static images) to obtain the baseline of scrotum temperature and 20 thermal recovery images were recorded after the cold stress to study the thermal recovery properties. Each image series was corrected for motion artifacts by means of a contour alignment algorithm. The cold stress was achieved by applying a dry patch—maintained at 10°C—to the scrotum for 2 min. The penis was protected from the cold stress by avoiding any possible contact with the cooling patch, which was shaped to be in contact with the scrotum only. Four regions of interest (ROIs) were selected for each of the two hemiscrota at both the testicle site and the pampiniform plexus. Re-warming curves (see Figure 1) were obtained separately, by averaging the temperature of the pixels within the cutaneous projection at each region of interest based on our previous study (Merla et al., 2002c). In particular, we have four ROIs: the first two located at the pampiniform plexus in the right and left hemiscrota (pp_r_) and (pp_l_, respectively), and the other two located at the testicular site in the right and left hemiscrota (ts_r_) and (ts_l_, respectively).

### 3.3. Direct estimation of the time-domain specifications

The scrotal thermoregulation system is a highly complex system that can be thought to connect some input variables (recorded by local and central thermal receptors) to the output variables constituted by the thermoregulatory effectors (Trafford et al., 1982). The wide number of complex processes potentially involved in temperature control and in its alteration suggest to consider the overall control system as a black box, whose overall structure can be investigated by analyzing the input-output time-responses (Trafford et al., 1982), either in the healthy and in the pathological conditions. Standard tools from control system theory can characterize the mathematical model of an unknown system by studying its dynamic response in the time-domain or, more specifically, by analyzing certain parameters characterizing the system response to canonical inputs (the step input, in our case). A first inspection of the healthy subjects data seemed to suggest that the scrotal thermoregulatory system could be described as a second-order time-invariant system with exponential decay (Merla et al., 2002b,c). An analogous model seemed able to describe responses obtained with varicocele patients, given that the scrotum affected by varicocele recovers faster from a controlled cold stress than healthy one (Merla et al., 2002c).

The different dynamics found in the two categories of subjects could be linked to the functional and morphological alterations associated with the presence of the disease. When trying to mathematically estimate the time domain parameters for each study group, it was noticed that the transient response of HCS curves has no overshoot, thus suggesting that a simple second-order model with unitary damping could be enough to describe the recorded responses. The same model was used for the vr1, vr2, and vr3 groups. The position of the coincident real poles characterizing the time behavior in the Complex plane can be estimated using the so-called Rise Time (*t*_r_), i.e., the time required for the step response to rise from 10 to 90% of its final value, suggesting closed loop poles (*S*_1_ and *S*_2_) given by Golnaraghi and Kuo (2010):

(1) $$S_{1},S_{2}=\frac{-1.8}{t_{\text{r}}}$$

In addition to closed loop poles, also the delay time (*t*_d_) (i.e., the time required for the step response to reach 50% of its final value) and the steady state error (*e*_ss_) (i.e., the discrepancy between the output and the reference input when the steady state is reached) have been considered for characterizing the recorded dynamic responses (Golnaraghi and Kuo, 2010). Such time-domain dynamic parameters are summarized in Table 4. Broadly speaking, the delay time td can be associated to the efficiency of the thermal exchange between the scrotal layer and the inner structures during the transient phase of rewarming. On the contrary, the rise time (*t*_r_) can be thought to represent the promptness of response of the thermal system to external and internal perturbations. As a whole, the closed loop poles *S*_1_ and *S*_2_ summarize the dynamic behavior of the system. Finally, the steady state error (*e*_ss_) determines the effectiveness of the feedback control action in achieving the steady state, restoring the reference basal conditions.

**Table 4:** **The transient and steady state characteristics of a control system in terms of the unit-step response**.

**Parameter symbol**	**Parameter name**	**Calculation description**
*t*_d_	Delay time	The delay time is calculated as the time required for the step response to reach 50"% of its final value (i.e., recovery point after 20 min)
*t*_r_	Rise time	The rise time is calculated as the time required for the step response to rise from 10 to 90 of its final value (i.e., recovery point after 20 min)
*e*_ss_	Steady state error	The steady-state error of a system response is defined as the discrepancy between the output and the reference input when the steady state (*t* → ∞) is reached. *e*_ss_ = reference input (baseline constant temperature value) − (final value, i.e., final recovery point after 20 min)
*S*_1_,*S*_2_	Closed loop poles	$S_{1},S_{2}=\frac{-1.8}{t_{\text{r}}}$

### 3.4. Data analysis

A Matlab (www.mathworks.com) home-made script was used for data and graphic analysis. The scrotal temperature T was measured both during the baseline and the 20 min after the cold challenge according to Mariotti et al. (2010). By using IR static images of the scrotum, the basal scrotal temperatures (T) has been measured and its average computed to provide the constant basal temperature values. For both the hemi-scrota, all of the parameters were computed at the level of pampiniform plexuses and at that of the testicles. All the temperature data were filtered through a smoothing algorithm (span = 5 samples). The statistical analysis was performed to search for differences in the time-domain parameters and scrotal temperature among HCS, vr1, vr2, and vr3. The distributions of the average parameters for each group were tested for normality by visual inspection of the frequency distribution and ShapiroWilk test. All the parameters for each group were compared through Wilcoxon-Mann-Whitney test (Glantz, 2005). The level of statistical significance was fixed at 0.01, resulting after performing Bonferroni correction by dividing the critical *p*-value (=0.05) by the number of comparisons being made (=4, i.e., number of compared groups HCS, vr1, vr2, and vr3 being considered in this study). A multiple logistic regression classification algorithm (Agresti, 2002) was performed in order to evaluate which parameter, or combination of parameters, better reproduces the probability to detect and classify the presence of varicocele with different grades (i.e., vr1, vr2, and vr3) as clinically evaluated through echo color Doppler. The clinical diagnosis was adopted as independent variable. The multi logistic regression is simply represented by Y = β X+ ε where Y is the matrix represented the dependent variables (which, in our case study, are the three varicocele groups vr1, vr2, vr3, initially diagnosed with respect to HCS using the standard echo Doppler test), X is design matrix of predictors (which, are the five extracted parameters, four time domain parameters *t*_d_, *t*_r_, *S*_1_ and *S*_2_ and *e*_ss_, and the basal temperature T), β is the matrix of the estimated variables that we need to estimate for the corresponding predictors to give the minimum values of error matrix ε. The cut-off for the best classification was established by means of a receiver operating characteristic (ROC) analysis (Westin, 2001) applied to the multiple logistic regression model output. ROC analysis allows the evaluation of the optimal cut-off for a binary classification resulting from a compromise between the 1-specificity, i.e., the false-positive rate, and the sensitivity, i.e., the true positive rate (Westin, 2001). In order to check which region of interest is the best region for classification among groups, the multinomial logistic regression approach were implemented five times, one time for the calculated time domain parameters from each region of interest i.e., pp_r_, ts_r_, pp_l_, and ts_l_ and one more time for the combining of both ts_l_ and pp_l_. ROC analysis was used to compare among their classification result based on the 1-specificity and the sensitivity.

## 4. Results

The distributions of the average parameters for each group rejected the null hypothesis of the normality test with significant level <0.01. For each region of interest, the group mean and the standard deviation (Std) for each parameter are reported in Table 5. Not all the estimated parameters for the right hemi-scrotum show statistical significant differences among groups (see Table 6). On the other hand, the estimated parameters for the left hemi-scrotum show statistical significant differences among groups, depending on the grade of the varicocele (see Table 6). The steady state error did not show any significant difference between groups, but for pp_l_, ts_l_, and ts_r_ (see Table 6). The rise time values measured at pp_l_ showed statistical significant differences (Table 6) for the vr2 and vr3 with respect to HCS, without any differences shown within varicocele groups. However, ts_l_ values showed statistical differences with respect to HCS only for vr1 and vr2. The closed-loop poles location values presented the same trend as the rise time. The only significant difference for the delay time (see Table 6) was for pp_r_ between vr1 and vr2. The basal temperature values for all patient groups are higher than that of the healthy group. The basal temperatures presented significant differences (see Table 6) between HCS and vr3 for all the scrotal regions; between vr1 and vr3 only for pp_r_ and pp_l_; between vr2 and vr3 only for pp_l_. The basal temperatures did not show any significant result between HCS and vr1 for all regions of interest.

**Table 5:** **Group subject average values at the regions of interest**.

Region of Interest (ROI)^*^	Group^**^	**Parameter (symbol) (unit if applicable)**				
		**Delay time (*t*_d_) (min)**	**Rise time (*t*_r_) (min)**	**Poles (*S*_1_, *S*_2_)**	**Error (*e*_ss_) (°C)**	**Temperature (*T*_b_) (°C)**
pp_r_	HCS	2.6 (0.4)	7.7 (0.9)	0.24 (0.02)	21.1 (1.5)	30.7 (1.1)
	vr1	2.5 (0.4)	6.8 (1.3)	0.27 (0.01)	21.1 (1.5)	30.9 (1.2)
	vr2	2.7 (0.5)	7.7 (1.1)	0.27 (0.06)	22.2 (1.5)	30.9 (0.9)
	vr3	2.7 (0.5)	7.3 (1.3)	0.26 (0.05)	22.1 (1.9)	31.5 (1.3)
ts_r_	HCS	2.7 (0.5)	7.8 (0.8)	0.23 (0.02)	20 (1.49)	30.6 (1.1)
	vr1	2.6 (0.5)	7.1 (1.1)	0.26 (0.05)	21 (1.3)	30.8 (1.1)
	vr2	2.8 (0.5)	7 (1.3)	0.27 (0.11)	22.2 (1.6)	30.9 (1.1)
	vr3	2.7 (0.6)	7.5 (1.5)	0.25 (0.06)	22.4 (1.6)	31.1 (1.1)
pp_l_	HCS	2.7 (0.5)	8.1 (1)	0.22 (0.03)	20.9 (1.6)	30.9 (1.1)
	vr1	2.4 (0.4)	7.4 (1.4)	0.25 (0.05)	21.2 (1.8)	31.3 (1.1)
	vr2	2.9 (0.6)	7.1 (1.5)	0.27 (0.07)	22.4 (1.6)	31.7 (0.8)
	vr3	2.5 (0.5)	6.5 (1.9)	0.3 (0.1)	22.1 (2)	32.5 (1.2)
ts_l_	HCS	2.7 (0.4)	7.7 (1)	0.24 (0.04)	20.7 (1)	30.9 (0.9)
	vr1	2.6 (0.4)	7.4 (1.4)	0.25 (0.06)	21.3 (1.6)	30.93 (1)
	vr2	2.8 (0.5)	7.1 (0.6)	0.28 (0.1)	22.6 (2)	31.5 (0.9)
	vr3	2.7 (0.5)	7.1 (1.5)	0.27 (0.08)	22.4 (1.6)	31.77 (1)

* Abbreviation definition for the region of interest: pp_r_ used for pampiniform plexus in the right scrotum, ts_r_ used for testicular site in the right scrotum, pp_l_ used for pampiniform plexus in the left scrotum, ts_l_ used for testicular site in the left scrotum.

** Abbreviation definition for the group: HCS used for Healthy controls, vr1 used for grade I and II of varicocele, vr2 used for grade III of varicocele and vr3 used for grade IV and V of varicocele.

**Table 6:** **Wilcoxon test outcomes**.

**Parameter (Symbol)**	Group^***^	Region of Interest (ROI)^**^											
		**pp_r_**			**ts_r_**			**pp_l_**			**ts_l_**		
		***W***	***Z***	***P***	***W***	***Z***	***P***	***W***	***Z***	***P***	***W***	***Z***	***P***
Delay time (*t*_d_)	HCS-vr1	1000	1.4	>0.01	1000	0.6	>0.01	1000	1.8	>0.01	1000	0.9	>0.01
	HCS-vr2	977	−0.84	>0.01	976	−0.84	>0.01	921	−1.5	>0.01	930	−1.5	>0.01
	HCS-vr3	865	0.4	>0.01	828	−0.1	>0.01	765	−1.2	>0.01	893	0.8	>0.01
	vr1-vr2	949	−1.9	>0.01	977	−1.4	>0.01	851	−3.1	<0.01^*^	921	−2.3	>0.01
	vr1-vr3	931	1.2	>0.01	877	0.3	>0.01	871	0.2	>0.01	956	1.6	>0.01
	vr2-vr3	863	−0.4	>0.01	832	−0.9	>0.01	727	−2.4	>0.01	857	−0.6	>0.01
Rise time (*t*_r_)	HCS-vr1	1000	0.7	>0.01	1000	2.2	>0.01	1000	2.2	>0.01	1198	2.8	<0.01^*^
	HCS-vr2	1000	1.5	>0.01	1000	−2.3	>0.01	1251	2.7	<0.01^*^	1262	2.9	<0.01^*^
	HCS-vr3	736	−1.6	>0.01	841	0.01	>0.01	629	−3.2	<0.01^*^	770	−1.1	>0.01
	vr1-vr2	1154	0.8	>0.01	1087	−0.01	>0.01	1000	0.6	>0.01	1046	−0.5	>0.01
	vr1-vr3	791	0.9	>0.01	954	1.5	>0.01	747	−1.6	>0.01	950	1.4	>0.01
	vr2-vr3	880	−0.2	>0.01	1023	1.8	>0.01	818	−1.1	>0.01	984	1.2	>0.01
Poles (*S*_1_,*S*_2_)	HCS-vr1	939	−0.7	>0.01	833	−2.2	>0.01	834	−2.2	>0.01	786	−2.8	<0.01^*^
	HCS-vr2	923	−1.1	>0.01	860	−2.3	>0.01	826	−2.7	<0.01^*^	815	−2.9	<0.01^*^
	HCS-vr3	943	1.5	>0.01	839	0.01	>0.01	1051	3.2	<0.01^*^	910	1.1	>0.01
	vr1-vr2	1022	−0.8	>0.01	1089	0.01	>0.01	1000	−0.66	>0.01	1130	0.5	>0.01
	vr1-vr3	917	0.9	>0.01	754	−1.5	>0.01	960	1.6	>0.01	757	−1.4	>0.01
	vr2-vr3	911	0.2	>0.01	769	−1.8	>0.01	974	1.1	>0.01	808	−1.2	>0.01
Steady state error (*e_ss_*)	HCS-vr1	929	−0.8	>0.01	998	0.07	>0.01	969	−0.3	>0.01	997	0.06	>0.01
	HCS-vr2	620	−4.5	<0.01^*^	759	−3.6	<0.01^*^	774	−3.4	<0.01^*^	802	−3	<0.01^*^
	HCS-vr3	1100	3.9	<0.01^*^	1072	3.5	<0.01^*^	1008	2.5	<0.01^*^	990	2.3	>0.01
	vr1-vr2	810	−3.4	<0.01^*^	830	−3.2	<0.01^*^	822	−3.3	<0.01^*^	821	−3.3	<0.01^*^
	vr1-vr3	1056	2.9	<0.01^*^	1079	3.3	<0.01^*^	1013	2.3	>0.01	1025	2.5	<0.01^*^
	vr2-vr3	874	−0.2	>0.01	948	0.7	>0.01	868	−0.4	>0.01	899	0.03	>0.01
Basal temperature (*T_b_*)	HCS-vr1	974	−0.2	>0.01	903	−1.2	>0.01	905	−1.2	>0.01	931	−0.8	>0.01
	HCS-vr2	891	−1.8	>0.01	950	−1.1	>0.01	821	−2.8	<0.01^*^	955	−1.1	>0.01
	HCS-vr3	1018	2.7	<0.01^*^	1120	2.6	<0.01^*^	1121	4.3	<0.01^*^	1007	2.5	<0.01^*^
	vr1-vr2	944	−1.8	>0.01	1093	0.06	>0.01	953	−1.6	>0.01	1078	−0.1	>0.01
	vr1-vr3	1038	2.7	<0.01^*^	957	1.5	>0.01	1090	3.4	<0.01^*^	978	2.1	>0.01
	vr2-vr3	984	1.2	>0.01	1009	1.6	>0.01	1079	2.5	<0.01^*^	1050	2.1	>0.01

* Means statistically significant.

** Abbreviation definition for the region of interest: pp_r_ used for pampiniform plexus in the right scrotum, ts_r_ used for testicular site in the right scrotum, pp_l_ used for pampiniform plexus in the left scrotum, ts_l_ used for testicular site in the left scrotum.

*** Abbreviation definition for the group: HCS used for Healthy controls, vr1 used for grade I and II of varicocele, vr2 used for grade III of varicocele and vr3 used for grade IV and V of varicocele.

Table 7 illustrates the sensitivity and 1-specifity value from the ROC analysis for each of the five multinomial logistic regression models adopted. The comparison of the Roc analysis results showed that the best sensitivity and the 1-specifity values for classification among groups were achieved by considering both pp_l_ and ts_l_. Based on the chosen time-domain parameters, we got three multiple logistic regressions equations with respect to HCS: the first for vr1, the second for vr2, and the third for vr3. Table 8 reports the classification discriminant parameters of the estimation of the predictor coefficient (β) with its SE, the Wald Statistics and the odds ratio of response variable (Exp(β)) with respect to the predictor coefficients. Positive value of the predictor coefficient indicates that higher values for the calculated parameters are related to a higher probability of positive diagnosis at the echo color Doppler investigation. The Wald Statistics validates the correlation between the calculated parameters and the presence of left varicocele.

**Table 7:** **ROC Cut-off thresholds**.

Region of Interest (ROI)^*^	Group^**^	**Cut-off**	**Senstivity**	**Specifity**
pp_r_	HCS-(vr1,vr2,vr3)	0.21	0.9032	0.4421
	vr1-(vr2,vr3)	0.19	0.8438	0.4444
	vr2-vr3	0.19	0.7714	0.8929
ts_r_	HCS-(vr1,vr2,vr3)	0.21	0.8710	0.5158
	vr1-(vr2,vr3)	0.18	0.8125	0.5079
	vr2-vr3	0.22	0.8571	0.6429
pp_l_	HCS-(vr1,vr2,vr3)	0.2	0.8710	0.4
	vr1-(vr2,vr3)	0.2	0.9063	0.3651
	vr2-vr3	0.16	0.9143	0.6786
ts_l_	HCS-(vr1,vr2,vr3)	0.17	0.8710	0.4842
	vr1-(vr2,vr3)	0.23	0.75	0.4127
	vr2-vr3	0.24	0.8000	0.5000
pp_l_ + ts_l_	HCS-(vr1,vr2,vr3)	0.18	0.9032	0.2947
	vr1-(vr2,vr3)	0.18	0.9032	0.1429
	vr2-vr3	0.18	0.9032	0.2222

* Abbreviation definition for the region of interest: pp_r_ used for pampiniform plexus in the right scrotum, ts_r_ used for testicular site in the right scrotum, pp_l_ used for pampiniform plexus in the left scrotum, ts_l_ used for testicular site in the left scrotum.

** Abbreviation definition for the group: HCS used for Healthy controls, vr1 used for grade I and II of varicocele, vr2 used for grade III of varicocele and vr3 used for grade IV and V of varicocele.

**Table 8:** **Classification Discriminant parameters**.

**Equation**	ROI^**^	Parameter^***^	**β**	**Standard Error SE**	**Wald T stats**	**Df**	**Sig**	**Exp(β)**
HCS-vr1^*^	pp_l_	Intercept	−61.8	34.9	−1.8	1	0.07	0.0
		*t*_d_	−0.1	0.8	−0.1	1	0.9	0.9
		*t*_r_	0.6	1.3	0.5	1	0.6	1.8
		*S*_1_,*S*_2_	35.7	37.6	1	1	0.3	10^15^
		*e*_ss_	0.3	0.4	0.8	1	0.4	1.4
		*T*_b_	0.9	0.6	1.5	1	0.1	2.5
	ts_l_	*t*_d_	−0.4	0.9	−0.4	1	0.6	0.7
		*t*_r_	1.4	1.8	0.8	1	0.4	4
		*S*_1_,*S*_2_	60	51	1.2	1	0.2	10^26^
		*e*_ss_	−0.5	0.4	−1.3	1	0.2	0.6
		*T*_b_	0.03	0.5	0.1	1	0.95	1.0
HCS-vr2^*^	pp_l_	Intercept	−76	36.6	−2.1	1	0.03	0.0
		*t*_d_	2.3	0.9	2.7	1	0.01	10
		*t*_r_	0.7	1.3	0.5	1	0.6	1.9
		*S*_1_,*S*_2_	47	38.5	1.2	1	0.2	10^2^
		*e*_ss_	0.4	0.4	1.1	1	0.27	1.5
		*T*_b_	2.1	0.7	3.2	1	0.0	8.2
	ts_l_	*t*_d_	0.1	0.9	0.1	1	0.94	1.06
		*t*_r_	−0.04	1.9	−0.02	1	0.98	0.9
		*S*_1_,*S*_2_	38.2	52.4	0.7	1	0.46	10^26^
		*e*_ss_	−0.1	0.4	−0.22	1	0.8	0.9
		*T*_b_	−0.9	0.5	−1.8	1	0.06	0.4
HCS-vr3^*^	pp_l_	Intercept	−125	36	−3.4	1	0.0	0.0
		*t*_d_	0.84	0.9	0.9	1	0.3	2.3
		*t*_r_	0.9	1	0.7	1	0.4	2.5
		*S*_1_,*S*_2_	52.4	38.6	1.4	1	0.17	10^22^
		*e*_ss_	0.14	0.4	0.4	1	0.7	1.2
		*T*_b_	2.5	0.7	3.6	1	0.03	11.9
	ts_l_	*t*_d_	−0.7	1	−0.7	1	0.5	0.5
		*t*_r_	2.5	1.8	1.4	1	0.17	12.6
		*S*_1_,*S*_2_	82	52	1.6	1	0.11	10^26^
		*e*_ss_	0.1	0.4	0.2	1	0.8	1.1
		*T*_b_	−0.6	0.5	−1	1	0.26	0.5

* Abbreviation definition for the group: HCS used for Healthy controls, vr1 used for grade I and II of varicocele, vr2 used for grade III of varicocele and vr3 used for grade IV and V of varicocele.

** Abbreviation definition for the region of interest: pp_l_ used for pampiniform plexus in the left scrotum, ts_l_ used for testicular site in the left scrotum.

*** Abbreviation definition for the parameters: t_d_ used for the delay time, t_r_ used for the rise time, S_1_, S_2_ used for the closed loop poles, e_ss_ used for the steady state error and T_b_ used for the basal temperature.

Table 9 illustrates the total-group classification and the group-specific classification, respectively. The cut-off thresholds used within the patient-group classification are shown in Table 10. The specific-classification result shows: 90.32% ratio of the true-classified HCS; 94.44% ratio of the true-classified vr1 patients from vr3 group; 87.5% ratio of the true-classified vr3 patients from varicocele groups; and 86.67% ratio of the true-classified vr2 patients from vr1 groups.

**Table 9:** **Confusion matrix for group specific classification**.

Groups^**^	**Original group**	**Predicted group**				**Correctly classified%**
		**HCS**	**vr1**	**vr2**	**vr3**	
HCS-vr	HCS	28	3^*^			90.32
	vr1	14	18	–	–	56.25
	vr2	10	–	25	–	71.43
	vr3	4	–	–	24	85.71
	Healty- vr Classification Result					75.92
HCS-vr3	HCS	28	–	3^*^		90.32
	vr3	4	–	–	24	85.71
	Healty- vr3 Classification result					75.92
HCS-vr2,vr3	HCS	28	–	3^*^		90.32
	vr2	10	–	25	–	71.43
	vr3	4	–	–	24	85.71
	Healty- vr2, vr3 Classification result					82.49
vr3-vr1,vr2	vr1	14	17	–	1	94.44
	vr2	10	–	15	10	60
	vr3	4	3^*^		21	87.50
	vr3- vr1,vr2 Classification result					80.65
vr2-vr1	vr1	14	10	7	1	58.82
	vr2	10	2	13	10	86.67
	vr2-vr1 Classification result					72.75

* Means the corresponding merged groups.

– Means not included group.

** Abbreviation definition for the group: HCS used for Healthy controls, vr used for all varicocele grades, vr1 used for grade I and II of varicocele, vr2 used for grade III of varicocele and vr3 used for grade IV and V of varicocele.

**Table 10:** **ROC Cut-off thresholds used for patient classification**.

Groups^*^	**Cut-off**	**Sensitivity**	**Specifity**
vr3-vr1, vr2	0.25	0.875	0.255
vr2-vr1	0.29	0.866	0.411

* Abbreviation definition for the group: vr1 used for grade I and II of varicocele, vr2 used for grade III of varicocele and vr3 used for grade IV and V of varicocele.

## 5. Discussion

Varicocele is a widely spread male disease, which remains the leading correctable cause of male infertility (Kaufman and Nagler, 1986). Although the assessment of varicocele is usually performed by evaluating the venous blood reflux by using echo color Doppler (Pauroso et al., 2011), the functional assessment of alterations in scrotal thermoregulation could provide new insights about its pathophysiology. Thermal infrared imaging has proved to be useful in highlighting the functional contents of scrotal skin thermal distribution (Merla et al., 2002b). Several IR studies have been run, proposing thermography parameters in the assessment of varicocele; however it ended without any consensus regarding the description of the alterations of the scrotal thermoregulation likely associated with the disease. Therefore, the aim of this study was to identify effective parameters, which describe the functional differences shown by healthy and patients in the scrotal thermal recovery from a controlled thermal stress.

In this study, the scrotal thermoregulation system was analyzed using standard time-domain dynamic parameters of the proto-type second order control system (delay time, rise time, poles locations, and the steady state error) and the static basal temperature values, since it has been already proved that patients exhibit different thermoregulatory dynamic responses with respect to HCS to the external thermal stimuli (Mariotti et al., 2010). Alterations in thermoregulation system may be due to the imbalance between vasoconstriction and vasodilation of the peripheral blood flow (Sanial and Maji, 2001). Owing to the complexity of the system, it is extremely difficult to isolate each specific individual function responsible for the impairment (i.e., the thermo-reception or the peripheral responses), and so it is necessary to consider the system as a black box which cannot be opened, but whose functions can be deduced from the analysis of the relations between the disturbances and responses (Sanial and Maji, 2001). In the theory of the control systems, the dynamic response of the scrotal system can be studied by analyzing its time-domain specifications (Golnaraghi and Kuo, 2010). Employing the time-domain analysis, both patient and healthy groups appear to exhibit the critically damping dynamic responses (Rene et al., 1995; Merla et al., 2002b), which are a special case of the prototype second order control system (Golnaraghi and Kuo, 2010). Consequently, from a theoretical point of view, to have a rigorous standard criterion to investigate the differences in the thermoregulation systems of patients and healthy groups, we suggested to adopt the critically damping prototype second-order model and try to mathematically estimate its time-domain parameters in order to classify among groups. In this article, we introduced the delay time to describe the altered recovery during the transient phase of warming and the rise time to examine the performance of the thermal recovery response to the external and internal stimuli. The closed-loop poles location measures the efficiency and stability performance. The steady state error parameter studies the ability of the feedback control system in restoring and maintaining the reference basal conditions (Mariotti et al., 2010).

Our proposal for time-domain analysis confirmed the previous experimental evidence (Shitzer et al., 1996) that the scrotal thermoregulatory response after the cooling is instantaneous. In fact, the recovery patterns did not show any lag time from the onset of the warming process and the end of the exposure to the cold (the temperatures increases immediately after the removal of cooling patch). Please note that there is a difference between the meanings of the lag time and of the delay time, as the lag time measures the delaying time before the onset of the recovery, while the delay time measures the delaying time during the transient phase of recovery. The delay time did not show any significant differences among all of the patient groups and the healthy group. On the other hand, our results suggest that varicocele induces faster warming by exerting higher rate of heat exchange between the cutaneous layer and the inner structures during the steady state phase of the recovery (Mariotti et al., 2010). This is probably due to the increased core body temperature contributed by testes where the cooling mechanism of counter-current heat exchange in the pampiniform plexus becomes defective because of the venous stasis associated to the disease (Sorensen et al., 1991). In fact, the group-average values of the rise time measured at pp_l_, ts_l_, pp_r_, and ts_r_, were lower than for healthy groups for all of the varicocele groups (Table 5). In addition, we found that the group-average values of the closed loop poles increase as the grade of varicocele becomes higher, elucidating that the defective thermal conditions associated with varicocele become more chronically stable in the later stages of the disease, emphasizing thus the importance of primary treatment of the disease. vr2 and vr3 patients presented higher *e*_ss_ values than HCS and vr1 (Table 5). This finding suggests that the higher stages of varicocele impair the feedback control role of the thermoregulation system in restoring and in maintaining the basal scrotal temperature after the cooling. Consequently, this result confirms the previous studies that concluded that, in abnormal situations with impaired anterior-venous testicular systems, there may be chronic dys-regulation, which may result in substantial changes in scrotal temperatures (Thonneau et al., 1998). Contrarily to Mariottis conclusions (Mariotti et al., 2010), this finding showed that the active processes of thermoregulatory vasodilation induced by cold stress do not act similarly in the presence and absence of the disease, as proven by *e*_ss_ values significantly different between healthy subjects and varicocele patients with higher disease grades. vr1 exerted healthy thermoregulation, as there was no significant difference between HCS and vr1. In addition, the smallest *e*_ss_ values measured in HCS and vr1 indicate that in the healthy or at the early stage of the disease scrotum the external cooling activates a feedback mechanism to control the temperature and to restore the basal conditions. This is opposite to the earlier suggestion of no scrotal feedback thermoregulation in humans (Sealfon and Zorgniotti, 1991). The chronic thermal dys-regulation associated with varicocele could explain the higher recovery temperatures, especially by the left scrotum (Figure 1). Moreover, the higher basal temperature in case of varicocele, mostly for vr3, characterized both the testicle and the pampiniform plexus of both hemi-scrota. This result could be explained as the dilatation of testicular veins due to venous incompetence in the pampiniform plexus (and/or in the spermatic veins), which reduces the venous return in the scrotum, causing a stagnation of flood and venous hypertension, and edema, thus increasing the testicular temperature (Ledda et al., 1996). Moreover, this observation suggests to further investigate possible relationships between abnormal thermoregulation and spermatogenesis (Kaufman and Nagler, 1986; Mieusset and Bujan., 1995; Merla et al., 2002c; Mariotti et al., 2010).

The Experimental data used in the present paper reported that all the computed parameters at the level of pampiniform plexuses and that of the testicle in the left scrotum (pp_l_ + ts_l_) could provide better diagnosis of the varicocele patients with respect to healthy subjects than right sided ones This result emphasizes on the importance of studying the left scrotum hyperthermia in the assessment of varicocele (Tucker, 2000; Merla et al., 2001, 2002b,c, 2004; Nogueira et al., 2009; Kulis et al., 2012a,b).

Three out of the healthy controls were misclassified as varicocele patients. On the other hand, we found an higher numbers of patients misclassified as healthy subjects. In particular, the ratio of misclassified patients decreases with the increased severity of the disease (Table 9). This result came as we expected. In fact, the higher the level of varicocele, the greater the scrotal thermoregulation impairment due to the higher induced vasodilation with respect to normal situations. However, the reasons for these misclassifications remain to be further clarified. The main target of the present study was to find effective parameters in the assessment of the thermal impairment secondary to varicocele. The using of the control system theory to characterize the time-domain response opens new perspectives of research for studying possible hemodynamic alterations associated with varicocele and its pathophysiology. In fact, the presented results suggest that the scrotal cutaneous hyperthermia secondary to varicocele was not only attributable to larger rates of convective exchange within the inner structures, but it could also be attributable to the active processes of vasodilation induced by the increased blood reflux. The application of this approach could help to understand which specific functional level of impairment may impact spermatogenesis. However, the proposed technique does not replace the gold standard tests that are usually performed, but it has to be considered as complementary to those or as an integrative method to be used for a more complete analysis. We found that the method of direct estimation of the closed loop dynamic response parameters based on time-domain analysis could: (1) become an useful tool in studying the physiological control systems of the human body with respect to a given disease; (2) provide an easy and quick numerical assessment of the disease process, since the values of the parameters can be calculated directly from the temperature curves. However, it should be pointed out that this method is limited at studying a well-known step response and that the time-domain specifications are applicable only for systems lower than the third order (Golnaraghi and Kuo, 2010). So, the user must be careful with respect to the order of the standard system used and to which standard input signal will be applied to study the dynamic performance of that system. For future work, the next step will be to study the functional correlation with spermatogenic data taken from the patients, to assess which of functional impairment on the spermatogenesis could depend on the impaired thermoregulation.

### Conflict of interest statement

The authors declare that the research was conducted in the absence of any commercial or financial relationships that could be construed as a potential conflict of interest.
